# Comparison of NGS panel and Sanger sequencing for genotyping CAG repeats in the *AR* gene

**DOI:** 10.1002/mgg3.1207

**Published:** 2020-03-25

**Authors:** Maria Santa Rocca, Margherita Ferrarini, Aichi Msaki, Cinzia Vinanzi, Marco Ghezzi, Maurizio De Rocco Ponce, Carlo Foresta, Alberto Ferlin

**Affiliations:** ^1^ Unit of Andrology and Reproductive Medicine Department of Medicine University of Padua Padua Italy; ^2^ Department of Clinical and Experimental Sciences University of Brescia Brescia Italy

**Keywords:** androgen receptor, HipSTR, NGS panel, Sanger, STR

## Abstract

**Background:**

The androgen receptor (AR) is a nuclear receptor, encoded by the *AR* gene on the X chromosome. Within the first exon of the *AR* gene, two short tandem repeats (STR), CAG and GGC, are a source of polymorphism in the population. Therefore, high‐throughput methods for screening *AR*, such as next‐generation sequencing (NGS), are sought after; however, data generated by NGS are limited by the availability of bioinformatics tools. Here, we evaluated the accuracy of the bioinformatics tool HipSTR in detecting and quantify CAG repeats within the *AR* gene.

**Method:**

The *AR* gene of 228 infertile men was sequenced using NGSgene panel. Data generated were analyzed with HipSTR to detect CAG repeats. The accuracy was compared with the results obtained with Sanger.

**Results:**

We found that HipSTR was more accurate than Sanger in genotyping normal karyotype men (46,XY), however, it was more likely to misidentify homozygote genotypes in men with Klinefelter syndrome (47,XXY).

**Conclusion:**

Our findings show that the bioinformatics tool HipSTR is 100% accurate in detecting and assessing *AR* CAG repeats in infertile men (46,XY) as well as in men with low‐level mosaicism.

## Graphical abstract

HipSTR was used as bioinformatics tool to detect CAG repeats. HipSTR genotyped men with normal karyotype (46,XY) with a higher accuracy compared with Sanger.

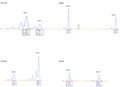

## 1 INTRODUCTION

The androgen receptor (AR) (MIM: *313700) belongs to the ligand‐activated nuclear receptor superfamily of transcription factors (Davey & Grossmann, 2016).

The main ligands that activate the AR are testosterone and dihydrotestosterone. The AR regulates a plethora of genes important for sex development (Hiort, 2013). The AR gene maps on X chromosome at Xq11.2‐12 and the human AR protein consists of 919 amino acids. Genetic variations in this gene have been associated with several pathological conditions such as Androgen Insensitivity Syndrome (MIM: #300068), Kennedy spinal and bulbar muscular atrophy (MIM: #313200), as well as several cancers (Garolla et al., 2005; Giovannelli et al., 2018; Lallous et al., 2016). Given the strong role of the AR in several diseases, a free online database dedicated to all known AR mutations is available since 1994 (http://androgendb.mcgill.ca/) (Gottlieb, Beitel, Nadarajah, Paliouras, & Trifiro, 2012; Patterson, Hughes, Gottlieb, & Pinsky, 1994). Reported genetic variations in AR include single‐point mutations, short insertions/deletions (indels), and splice site mutations (Shukla, Plaga, Shankar, & Gupta, 2016). Another source of polymorphism occurs in two distinct short tandem repeats (STRs) located in exon 1. These trinucleotide repeats, CAG and GGC, are translated into polyglutamine and polyglycine stretches in the transactivation domain of the AR protein (Chamberlain, Driver, & Miesfeld, 1994; Claessens et al., 2008; Need et al., 2009).

These repeats can vary in length and show remarkable ethnic differences (Lund, Tapanainen, Lähdetie, Savontaus, & Aittomäki, 2003). Caucasians have an average of 21–22 CAG repeats and 17–18 GGC repeats (Edwards, Hammond, Jin, Caskey, & Chakraborty, 1992; Ferlin et al., 2004; Platz et al., 1998; Zitzmann & Nieschlag, 2003). It is noteworthy that GGC repeats are generally less polymorphic than CAG repeats (Stanford et al., 1997).

While it is well established that CAG repeats length affects AR transcriptional activity (Tirabassi et al., 2015), it is not known how GGC repeat variations affect AR function (Ferlin et al., 2005). Some studies found that short GGC repeats associated with cancer and male infertility (Ding, Xu, Menon, Reddy, & Barrack, 2005; Ferlin et al., 2004; Sasaki et al., 2005).

Inversely, longer CAG repeats have been associated with male and female infertility (Ashraf, Tariq, & Rehman, 2019; Mobasseri, Babaei, Karimian, & Nikzad, 2018; Xiao et al., 2016). Klinefelter's syndrome is defined by a supernumerary X chromosome (47, XXY) and is the most common genetic cause of male hypogonadism and infertility (Aksglaede et al., 2013; Ferlin et al., 2019; Rocca et al., 2016). Men with this syndrome have various degrees of physiological and intellectual disabilities with the severity depending on the expression of the genetic defect.

Although the extra X chromosome in KS men is inactivated just as it occurs in women, a preferential inactivation of the X chromosome carrying AR allele with longer CAG stretch has been reported in some cases, Suzuki et al. (2001) whereas others have found the contrary to be true (Zitzmann, Depenbusch, Gromoll, and Nieschlag 2004). To date, there is no agreement on which of the two alleles is preferentially expressed.

Women carrying AR allele with long CAG repeats in the active X chromosome show a high risk of developing breast cancer, likely due to a nonrandomly X inactivation (Chen, Wu, Chen, Tsai, & Chien, 2014). Increased frequency of breast cancer is also more common in Klinefelter men (De Sanctis, Fiscina, Soliman, Giovannini, and Yassin 2013). CAG repeats, therefore, have been extensively studied in KS subjects in order to better understand their clinical features (Ferlin et al., 2011; Zinn et al., 2005; Zitzmann et al., 2004).

Overall, the accurate determination of CAG repeats in AR of infertile men, including Klinefelter, should be recommended in clinical practice as it can also predict the risk of developing several tumors (Ferlin et al., 2007; Garolla et al., 2005; Mao et al., 2015).

To date, polymerase chain reaction (PCR) is considered the gold standard method to investigate short tandem repeats and the resulting amplicons are resolved by several molecular technologies. The advancement of sequencing technologies has permitted the fast processing of multiple samples in the detection of single nucleotide variants including the expansion of short tandem repeats. Several analytical methods have evolved in order to determine STR detected by next‐generation sequencing (NGS) (Bahlo et al., 2018; Liu, Zhang, Wang, Gu, and Wang, 2017).

Here, we applied HipSTR as a bioinformatics method to assess CAG expansion within AR of 228 men analyzed by NGS and compared it with Sanger.

## 2 MATERIAL AND METHODS

### 2.1 Subjects

This study was approved by the hospital ethics committee and included 228 subjects retrospectively selected among men referred for fertility evaluation to our Centre (114 men with KS [109 nonmosaic 47,XXY and 5 mosaic 47,XXY/46,XY] and 114 nonsyndromic men [46,XY]). All subjects were of Caucasian ethnicity and Italian origin according to self‐report. Men with bone marrow transplant were excluded.

### 2.2 Amplification and allele sizing

Genomic DNA was extracted from peripheral blood leukocytes using QIAamp DNA Blood Mini Kit according to the manufacturer's protocol (Qiagen Inc.). The quality of the DNA was examined on a NanoDrop spectrophotometer (Thermo Fisher Scientific Inc).

**FIGURE 1:**
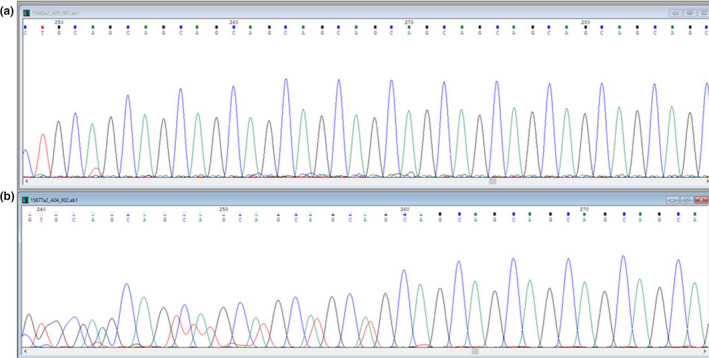
Representative Sanger chromatogram of *AR* CAG repeat region in a 46,XY and a Klinefelter 47,XXY men (a) Sanger chromatogram of a male with normal karyotype (46,XY) (b) Sanger chromatogram of a male with Klinefelter syndrome (47,XXY)

Determination of the CAG repeat number on *AR* gene was performed by Sanger as previously described (Ferlin et al., 2004). Sanger sequences (Figure 1) were analyzed with the gap4 software of the Staden package (Staden, 1996) available at the UK Human Genome Mapping Project webpage (http://www.hgmp.mrc.ac.uk/).

As an independent method to evaluate CAG repeat length, microsatellite analysis was performed. Briefly, the amplification of CAG polymorphism was performed in a 25‐µl PCR mixture containing 50 ng of DNA, 1 mmol/L each primer: 5’ end fluorescently labeled with carboxyfluorescein dye (FAM) forward primer 5’‐GTGCGCGAAGTGTCCAGAA‐3’, with its and unlabeled reverse primer 5’‐TAGCCTGTGGGGCCTCTACG‐3’ (Ackerman et al., 2012). The PCR mixture contained: PCR buffer, 80 uM dNTPs, 1mM MgCl2, and 1.0 U of Amplitaq Gold (Thermofisher) polymerase. Amplification was performed with an initial denaturation at 95°C for 5 min, followed by 35 cycles of denaturation at 94°C for 1 min, annealing at 53°C for 1 min, extension at 72°C for 1 min, and a final 15‐min extension at 72°C. The PCR fragments were resolved by electrophoresis on an automated ABI Prism 3130 XL Genetic Analyzer. GeneScan analysis was performed with PeakScan Software v.1.0 (Applied Biosystems).

### 2.3 Sequencing analysis by NGS panel

Sample libraries for sequencing were prepared starting from 80 ng of DNA using AmpliSeq Custom Panel (including probes for AR gene). Genes included in the custom panel were as follows: *AR (OMIM:* * *313700, NM_000044)* and TEX11 (OMIM *300311, NM_001003811) on X chromosome and *FSHR (OMIM:* * 136435, NM_000145), FSHB (OMIM: *136530, NM_001018080), KLHL10 (OMIM: *608778, NM_152467), NR5A1 (OMIM: *184757, NM_004959), NANOS1 (OMIM: *608226, NM_199461), SEPT12 (OMIM: *611562, NM_144605), and SYCP3 (OMIM: *604759, NM_001177948) on autosomal chromosomes. The libraries were generated using Amplification Library PLUS (24 Reactions) for Illumina according to the manufacturer's protocol (Illumina). The libraries were then loaded on a 500‐cycle (2 × 250 paired ends) reagent cartridge (Illumina) and run on a MiSeq sequencer (Illumina).

For each run, the average depth was of ~100X horizontal coverage to allow for optimal variant calling. BAM files were obtained aligning 250 bp reads to the hg19 reference genome with bwa‐mem (v. 0.7.17) (Li & Durbin, 2009) and were sorted and indexed with samtools (v. 0.1.19) (Li et al., 2009). The GGC repeats were not sufficiently covered by design of the probes.

### 2.4 STRs genotyping with HipSTR

HipSTR requires a BED file compiled as follows:

**Table:** 

chrX	66,765,160	66,765,261	3	34	CAG

The genomic coordinates referred to hg19 were inserted in the first three columns. The motif length was specified in the fourth column. The reference allele length was reported in the fifth column. Finally, in the optional sixth column, the specific analyzed STR locus was named.

In the genome CAG repeats in AR actually go from 66,765,160 to 66,765,225. This is followed by a shorter CAG repeats (6XCAG) 18 base pairs downstream. This poses a problem in the correct alignment of reads in this region. Therefore, the main CAG repeat, the intervening bases, and the shorter CAG repeat were considered as a single STR. Consequently, the reference allele length of the combined STR is 34 (22 + 18/3 + 6).

The hg19 reference sequences of all chromosomes were downloaded from http://hgdownload.cse.ucsc.edu/goldenPath/hg19/bigZips/chromFa.tar.gz. Chromosome sequences were then concatenated into a single FASTA file and indexed using samtools (v. 0.1.19) (Li et al., 2009).

HipSTR (v. 0.6.2) was used with Mode 1 with the following parameters: ‐‐max‐str‐len 105 and ‐‐no‐rmdup. This last parameter is necessary when processing PCR‐amplified reads.

Samples from 46,XY and KS men were analyzed separately. The option ‐‐haploid‐chrs chrX was used for 46,XY men because they are haploid for the X chromosome.

HipSTR calls were filtered out if the posterior probability of unphased genotype (Q) was <0.90 and the number of valid reads used for sample's genotype (DP) was <15. The confidence of heterozygous calls was assessed using a combination of the AB and MALLREADS values.

## 3 RESULTS

### 3.1 46,XY men

Of 114 men analyzed, 103 samples passed quality filters. We found that genotypes matching between Sanger and HipSTR were 102 and that the only one discordant genotype was correctly called by HipSTR (Table 1). Therefore, the accuracy of Sanger resulted to be lower than HipSTR (102/103 = 0.99 and 103/103 = 1, respectively).

**TABLE 1:** Comparison between Sanger and HipSTR in detecting the genotype of 46,XY men and 47,XXY men

	Results of genotyping
*46,XY (n = 103)*	
Matching genotypes	**102**
Discordant genotypes	**1**
Correct Sanger genotypes	0
Correct HipSTR genotypes	1
*47,XXY (n = 109)*	
Matching genotypes	**57**
Discordant genotypes	**52**
Correct Sanger genotypes	39
Correct HipSTR genotypes	13

Matching and discordant sequences are in bold.

## 4 47,XXY men

Sequencing data from 114 men with 47,XXY karyotype were analyzed. A total of 109 passed quality filters and were further characterized. Capillary electrophoresis determined that 67 were heterozygotes and 42 were homozygotes for *AR* gene on X chromosome.

**FIGURE 2:**
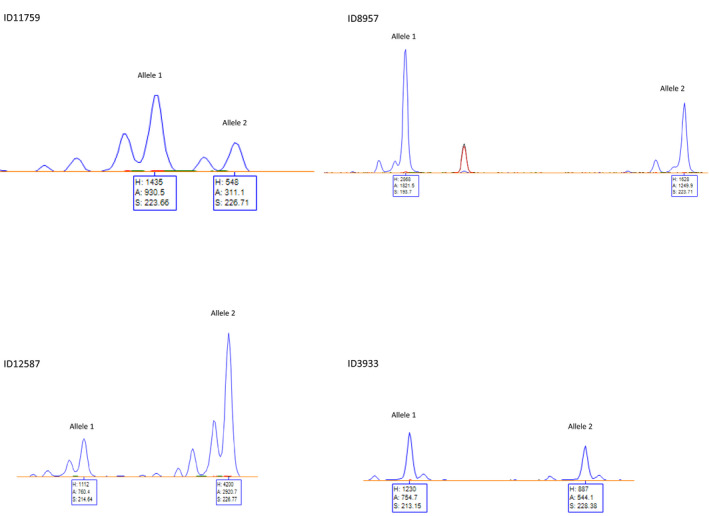
Microsatellite analysis of the *AR* CAG region of four Klinefelter patients in which genotyping was discordant between Sanger and HipSTR

We found that in 57 cases the Sanger genotype was in agreement with HipSTR. However, there were 52 discordant genotypes and according to capillary electrophoresis. According to microsatellite analysis, Sanger recognized correctly the genotype of 39 individuals, whereas HipSTR only recognized the genotype of 13 individuals (Table 1). Within these 13 cases, Sanger failed to recognize the heterozygosity of four individuals, namely, in the samples ID 3933, 8957, 12587, and 11759 (Figure 2) and the correct number of CAG repeats in nine cases (Table S1).

Therefore, the accuracy of Sanger and HipSTR resulted to be 88% (96/109) and 64% (70/109), respectively.

In Table 2 it is reported the ability of assigning the correct genotype, hence, the probability of identifying true heterozygous or homozygous genotypes. HipSTR identified all heterozygous genotypes, whereas Sanger was limited to 94%. In contrast, HipSTR correctly identified only 7% of the homozygote cases.

**TABLE 2:** Sensitivity of Sanger and HipSTR in detecting in 47,XXY subjects true heterozygotes and homozygotes identified by capillary electrophoresis

Method	Heterozygotes (*N* = 67)	Homozygotes (*N* = 42)
Sanger	94% (63/67)	100% (42/42)
HipSTR	100% (67/67)	7% (3/42)

## 5 DISCUSSION

This is the first study showing the STRs genotyping by HipSTR applied to a target NGS panel and evaluating its accuracy in comparison to Sanger for genotyping *AR* in 46,XY men and Klinefelter men.

STRs are nucleotide repeats spanning approximately 3% of the whole human genome (Dashnow et al., 2018). As expansions of nucleotide repeats can result in human diseases, the length determination of STR, mapping in coding or regulatory regions, is fundamental for the diagnosis of these pathologies (Paulson, 2018). The pathological STR expansion within *AR* gene leads to bulbospinal neuronopathy disorder.

In addition to this X‐linked neuropathy, the two polymorphic sites of *AR* gene are intensively studied as risk factors for infertility or cancer. Despite the high risk to miss heterozygosity due to a preferential amplification of one allele (Hamilton et al., 2016), Sanger method is generally used for genotyping *AR* gene.

Currently, Sanger has been increasingly supplanted by new high‐throughput technologies and the increasing progress of NGS has been followed by an equal progress in the bioinformatics field.

Although several bioinformatics tools are available for STRs analysis (Table 3), for this study we chose to use HipSTR (Willems et al., 2017) for the following reasons: (a) it estimates the allele sizes; (b) it has very high accuracy (Bahlo et al., 2018; Gymrek, 2017); (c) it allows a multisample analysis; (d) it analyzes exclusively Illumina data; and (e) it is able to manage differently diploid and haploid genotypes. The latter feature is ideal for genotyping STRs in sex chromosomes.

**TABLE 3:** Tools for genotyping STRs through NGS

Tool	Refs	Estimate STR length	Multisample analysis	Suitable for long STRs
lobSTR	Gymrek, Golan, Rosset, & Erlich (2012)	Y	Y	N
RepeatSeq	Highnam et al. (2013)	Y	N	N
HipSTR	Willems et al. (2017)	Y	Y	N
ExpansionHunter	Dolzhenko et al. (2017)	Y	N	Y
exSTRa	Tankard et al. (2018)	N	Y	Y
STRetch	Dashnow et al. (2018)	Y*	Y	Y

Note

Y* (not tested for PCR + or targeted sequencing data) (ref).

Abbreviations: N, no; NGS, next‐generation sequencing; STRs, short tandem repeats; Y, yes.

From the comparison between Sanger and HipSTR analysis, HipSTR resulted to be more accurate than Sanger in genotyping 46,XY men (100% vs. 94%), whereas it resulted less efficient than Sanger in genotyping 47,XXY males (64% vs. 88%). Indeed, we found that HipSTR frequently was not able to accurately distinguish homozygous genotypes, calling them as heterozygous genotypes whose two alleles differed for only one triplet. This error‐prone situation is likely due to PCR stutter products, however, it can be identified by the STR sizes given in the MALLREADS parameter (Willems et al., 2017).

Nevertheless, in four cases HipSTR prevailed over Sanger in accurately detecting the heterozygous genotypes, whose alleles differ, respectively, for one, two, four, and five triplets (Table S1, Figure 2). In these cases we found MALLREADS and AB values to be informative in clarifying the presence of two true alleles (Table S1).

Specifically, samples with ID 3933, 8957, and 12587 had AB values −0.1, −24.38, and −20.28, respectively, and these results would confirm that true heterozygous calls generally had AB values between 0 and −25. Moreover, for the same samples MALLREADS were indicative of heterozygosity as the most representative alleles differed by more than one triplet and, therefore, they were not consecutive. Interestingly, sample with ID 11759, missed by Sanger and showing a karyotype with 50% of mosaicism 47,XXY/46,XY, was correctly identified by HipSTR, despite it had AB value of −74.83. In this case, MALLREADS highlighted a highly probable second allele (Table S1).

The latter result underscores the risk of missing mild or low‐level mosaicism by Sanger. The frequency of mosaic forms is roughly 10%–20% in KS, but it is likely that this prevalence may be higher (Samplaski et al., 2014). Indeed, the broad spectrum of phenotypes in KS could depend on the presence or absence of mosaicism (46,XY/47,XXY) (Tüttelmann & Gromoll, 2010).

While the error of finding a heterozygous genotype with two alleles differing for a single triplet expansion might not be clinically relevant, in the case of the possible presence of low level of mosaicism could be worthwhile to further investigate cases that are not detected by traditional methods. In particular, results of HipSTR suggest that preferably 100 metaphases should be analyzed by karyotyping to exclude low level of mosaicisms.

Although Sanger and capillary electrophoresis are still the gold standard methods in detecting STR variation such as the CAG repeats in *AR* gene, the advent of NGS technology represents a big opportunity for investigating massively STR expansions. However, the short reads generated by the Illumina NGS panel are limited to 375 base pairs and, therefore, longer CAG expansions (beyond 30 repeats) could be not covered.

In conclusion, the evaluation of *AR* CAG repeats using bioinformatics tools in men with 47,XXY karyotype must be used with caution. However, we suggest to use NGS panel for the study of *AR* STR in 46,XY infertile men and men with a suspected low‐level mosaicism.

## CONFLICT OF INTERESTS

The authors declare that there is no conflict of interests.

## Supporting information

Table S1
