# Supplementary material for: Comparison of NGS panel and Sanger sequencing for genotyping CAG repeats in the AR gene
Source: Mol Genet Genomic Med. 2020 Mar 25;8(6):e1207. doi: 10.1002/mgg3.1207 (PMC7284049; doi:10.1002/mgg3.1207)
Supplement: Supplementary file 1 — Table S1 [file MGG3-8-e1207-s001.docx]

| **ID** | **(CAG)_n_ by Sanger** | **(CAG)_n_ by HipSTR** | **Capillary Electrophoresis** | **AB** | **MALLREADS** |
| --- | --- | --- | --- | --- | --- |
| 3933* | 24 | 19\|24 | 19\|24 | -0.10 | -27\|2;-24\|2;-15\|4;-12\|46;**-9\|140**;-6\|2;-3\|2;0\|18;3\|42;**6\|124** |
| 8957* | 22 | 22\|24 | 22\|24 | -24.38 | -15\|6;-9\|2;-6\|18;-3\|122;**0\|447**;3\|76;**6\|210**;9\|2 |
| 11759* | 22 | 22\|23 | 22\|23 | -74.83 | -18\|2;-12\|6;-9\|2;-6\|34;-3\|328;**0\|876**;**3\|454** |
| 12587* | 23 | 19\|23 | 19\|23 | -20.28 | -15\|8;-12\|54;**-9\|143**;-6\|4;-3\|24;0\|90;**3\|315**;6\|4 |
| 8144 | 24\|29 | 24\|28 | 24\|28 | -5.53 | -12\|2;-6\|2;-3\|4;0\|32;3\|161;**6\|406**;9\|8;12\|34;15\|208;**18\|529**;21\|6 |
| 10357 | 22\|27 | 22\|26 | 22\|26 | -7.13 | -18\|6;-15\|2;-9\|4;-6\|28;-3\|160;**0\|482**;3\|8;6\|33;9\|108;**12\|342** |
| 10938 | 24\|26 | 25\|27 | 25\|27 | -0.90 | -9\|9;-3\|4;0\|6;3\|50;6\|261;**9\|706**;12\|207;**15\|651**;18\|11 |
| 11851 | 18\|23 | 18\|22 | 18\|22 | -3.00 | -30\|4;-21\|2;-18\|28;-15\|146;**-12\|699**;-9\|9;-6\|32;-3\|175;**0\|527**;3\|2 |
| 11863 | 23\|28 | 23\|27 | 23\|27 | -0.81 | -12\|6;-9\|2;-6\|2;-3\|52;0\|294;**3\|716**;6\|20;9\|50;12\|255;**15\|676**;18\|5 |
| 11935 | 24\|25 | 23\|24 | 23\|24 | -31.02 | -6\|6;-3\|43;0\|193;**3\|620**;**6\|407** |
| 11953 | 23\|32 | 22\|31 | 22\|31 | -15.12 | -18\|4;-15\|2;-12\|10;-9\|14;-6\|62;-3\|368;**0\|1051**;3\|4;9\|4;12\|6;15\|6;18\|24;21\|72;24\|343;**27\|641**;30\|6 |
| 12158 | 26\|30 | 26\|31 | 26\|31 | -6.49 | -6\|2;0\|4;3\|2;6\|18;9\|117;**12\|331**;21\|27;24\|88;**27\|212**;30\|2 |
| 12222 | 12\|14 | 12\|22 | 12\|22 | -3.96 | -39\|2;-36\|4;-33\|131;**-30\|695**;-27\|2;-15\|2;-12\|4;-9\|4;-6\|17;-3\|143;**0\|504**;3\|6 |
| 7225 | 22\|23 | 22\|23 | 22\|23 | -44.71 | -15\|4;-12\|2;-9\|10;-6\|24;-3\|175;**0\|564**;**3\|282**;6\|6 |
| 7545 | 21\|24 | 21\|24 | 21\|24 | -2.24 | -21\|2;-15\|2;-12\|8;-9\|14;-6\|116;**-3\|327**;0\|28;3\|76;**6\|280** |
| 7814 | 20\|21 | 20\|21 | 20\|21 | -16.89 | -24\|2;-18\|2;-12\|10;-9\|134;**-6\|397**;**-3\|281**;0\|4;6\|2 |
| 7971 | 20\|22 | 20\|22 | 20\|22 | -6.14 | -24\|2;-18\|2;-15\|6;-12\|24;-9\|157;**-6\|520**;-3\|108;**0\|429**;12\|2 |
| 8210 | 23\|24 | 23\|24 | NA | -53.97 | -15\|4;-12\|4;-9\|2;-6\|6;-3\|20;0\|92;**3\|365**;**6\|985**;9\|8 |
| 8211 | 21\|24 | 21\|24 | 21\|24 | -7.24 | -18\|2;-12\|2;-9\|18;-6\|118;**-3\|359**;0\|28;3\|168;**6\|491** |
| 8346 | 27\|28 | 27\|28 | 27\|28 | -39.06 | -3\|4;0\|6;3\|6;6\|6;9\|23;12\|96;**15\|422**;**18\|1001**;21\|2 |
| 8430 | 18\|25 | 18\|25 | 17\|25 | -2.32 | -30\|2;-27\|2;-21\|2;-18\|16;-15\|120;**-12\|410**;-9\|2;-6\|4;-3\|2;0\|6;3\|16;6\|114;**9\|332**;12\|2 |
| 8592 | 21\|23 | 21\|23 | 21\|23 | 0.00 | -21\|10;-18\|4;-15\|4;-12\|2;-9\|48;-6\|205;**-3\|673**;0\|237;**3\|705**;21\|1 |
| 8696 | 21\|22 | 21\|22 | 21\|22 | -34.82 | -21\|2;-15\|2;-12\|6;-9\|55;-6\|381;**-3\|1221**;**0\|938**;3\|5;6\|2 |
| 8813 | 21\|22 | 21\|22 | 21\|22 | -14.45 | -15\|2;-12\|2;-9\|36;-6\|174;**-3\|608**;**0\|508**;3\|4 |
| 9250 | 20\|24 | 20\|24 | 20\|24 | -4.68 | -24\|2;-18\|2;-15\|6;-12\|52;-9\|201;-**6\|594**;-3\|4;0\|27;3\|167;**6\|480**;9\|4 |
| 9501 | 23\|26 | 23\|26 | 23\|26 | -0.26 | 0\|6;**3\|26**;6\|2;9\|8;**12\|27** |
| 9634 | 22\|23 | 22\|23 | 22\|23 | -21.12 | -18\|4;-12\|4;-9\|3;-6\|28;-3\|204;**0\|840**;**3\|661**;6\|2;21\|2 |
| 9641 | 21\|23 | 21\|23 | 21\|23 | -1.51 | -21\|2;-15\|2;-12\|6;-9\|40;-6\|250;**-3\|801**;0\|259;**3\|735**;6\|6 |
| 9773 | 23\|24 | 23\|24 | 23\|24 | -29.21 | -6\|8;-3\|49;0\|190;**3\|802**;**6\|512**;9\|20;12\|2 |
| 9776 | 15\|16 | 15\|16 | 15\|16 | -29.26 | -39\|2;-30\|4;-27\|22;-24\|181;**-21\|759**;**-18\|499** |
| 9877 | 21\|26 | 21\|26 | 21\|26 | -0.15 | -12\|2;-9\|16;-6\|110;**-3\|286**;3\|10;6\|16;9\|110;**12\|288**;15\|2 |
| 9889 | 22\|27 | 22\|27 | 22\|27 | -7.85 | -18\|2;-12\|8;-9\|20;-6\|52;-3\|265;**0\|757**;3\|2;6\|14;9\|42;12\|203;**15\|586**;18\|4 |
| 10188 | 20\|23 | 20\|23 | NA | -5.68 | -18\|6;-15\|2;-12\|16;-9\|169;**-6\|571**;-3\|30;0\|136;**3\|420**;6\|2;15\|2 |
| 10234 | 20\|22 | 20\|22 | NA | -11.12 | -21\|4;-18\|2;-15\|6;-12\|48;-9\|248;**-6\|799**;-3\|205;**0\|589**;3\|4 |
| 10295 | 20\|24 | 20\|24 | NA | -0.12 | -24\|2;-18\|2;-15\|6;-12\|25;-9\|169;**-6\|538**;-3\|6;0\|44;3\|167;**6\|504**;9\|8 |
| 10383 | 22\|23 | 22\|23 | NA | -29.99 | -18\|2;-15\|8;-12\|2;-9\|6;-6\|28;-3\|173;**0\|524**;**3\|320** |
| 10502 | 21\|25 | 21\|25 | NA | -14.31 | -21\|2;-18\|2;-15\|2;-12\|10;-9\|30;-6\|110;**-3\|349**;0\|7;3\|48;6\|208;**9\|515**;12\|5;27\|2 |
| 10517 | 22\|23 | 22\|23 | NA | -32.27 | -18\|4;-15\|2;-12\|2;-6\|32;-3\|261;**0\|911**;**3\|637**;6\|2 |
| 10526 | 20\|23 | 20\|23 | NA | -2.75 | -24\|8;-21\|2;-15\|24;-12\|55;-9\|319;**-6\|991**;-3\|52;0\|311;**3\|878**;6\|4 |
| 10543 | 21\|23 | 21\|23 | NA | -0.99 | -21\|2;-18\|2;-12\|4;-9\|26;-6\|96;**-3\|347**;0\|101;**3\|322**;6\|2 |
| 10650 | 20\|24 | 20\|24 | NA | -0.04 | -24\|2;-12\|14;-9\|78;**-6\|291**;0\|18;3\|52;**6\|311** |
| 10728 | 21\|25 | 21\|25 | 21\|25 | -1.04 | -21\|1;-18\|6;-15\|2;-12\|2;-9\|39;-6\|289;**-3\|854**;0\|22;3\|49;6\|329;**9\|864**;12\|2;15\|2 |
| 10973 | 21\|26 | 21\|26 | NA | -5.65 | -21\|4;-15\|2;-9\|6;-6\|92;**-3\|310**;0\|4;3\|6;6\|6;9\|70;**12\|198**;15\|2 |
| 11170 | 19\|21 | 19\|21 | NA | -4.45 | -27\|4;-18\|6;-15\|25;-12\|244;**-9\|889**;-6\|220;**-3\|768** |
| 11240 | 23\|26 | 23\|26 | NA | -3.88 | -15\|1;-9\|2;-6\|2;-3\|38;0\|172;**3\|532**;6\|38;9\|157;**12\|405**;15\|2 |
| 11241 | 22\|26 | 22\|26 | NA | -7.55 | -18\|1;-12\|2;-9\|4;-6\|10;-3\|151;**0\|526**;3\|2;6\|30;9\|106;**12\|368**;15\|4 |
| 11260 | 21\|25 | 21\|25 | NA | -5.18 | -21\|9;-15\|4;-9\|40;-6\|209;**-3\|683**;0\|10;3\|53;6\|243;**9\|751**;12\|90;27\|1 |
| 11362 | 19\|21 | 19\|21 | NA | -0.77 | -24\|2;-18\|10;-15\|30;-12\|256;**-9\|856**;-6\|255;**-3\|817** |
| 11671 | 17\|24 | 17\|24 | NA | -6.44 | -33\|2;-27\|2;-24\|6;-21\|20;-18\|244;**-15\|992**;-12\|16;-3\|8;0\|45;3\|283;**6\|692**;9\|2 |
| 11688 | 20\|21 | 20\|21 | NA | -93.04 | -24\|2;-18\|4;-15\|6;-12\|46;-9\|324;**-6\|1112**;**-3\|538**;0\|4;6\|4 |
| 11701 | 22\|28 | 22\|28 | NA | -4.92 | -15\|2;-12\|4;-9\|2;-6\|8;-3\|95;**0\|456**;3\|2;6\|2;9\|10;12\|12;15\|102;**18\|296**;21\|4 |
| 11731 | 18\|21 | 18\|21 | NA | -20.35 | -30\|6;-27\|2;-18\|26;-15\|208;**-12\|723**;-9\|56;-6\|312;**-3\|1047** |
| 11841 | 23\|25 | 23\|25 | NA | -11.11 | -12\|4;-9\|10;-6\|17;-3\|48;0\|333;**3\|939**;6\|273;**9\|740**;12\|4 |
| 11877 | 17\|23 | 17\|23 | NA | -0.91 | -27\|2;-24\|2;-21\|40;-18\|313;**-15\|1296**;-12\|10;-9\|4;-6\|2;-3\|103;0\|374;**3\|1079**;6\|4;12\|2 |
| 11932 | 19\|28 | 19\|28 | 19\|28 | -25.36 | -27\|6;-24\|3;-21\|10;-18\|8;-15\|26;-12\|150;**-9\|525**;-3\|2;0\|2;3\|6;6\|6;9\|10;12\|92;15\|312;**18\|754**;21\|4 |
| 11960 | 24\|26 | 24\|26 | NA | -1.24 | -12\|2;-3\|2;0\|28;3\|130;**6\|455**;9\|147;**12\|402**;15\|8 |
| 11961 | 21\|24 | 21\|24 | NA | -0.79 | -6\|2;**-3\|33**;0\|2;3\|4;**6\|42**;9\|2 |
| 12007 | 13\|23 | 13\|23 | NA | -12.65 | -39\|2;-36\|4;-33\|8;-30\|100;**-27\|685**;-24\|8;-3\|28;0\|124;**3\|389** |
| 12074 | 25\|26 | 25\|26 | NA | -28.68 | -9\|2;-3\|2;0\|4;3\|36;6\|152;**9\|522**;**12\|320**;15\|4 |
| 12090 | 20\|23 | 20\|23 | NA | -1.54 | -24\|2;-21\|2;-15\|2;-12\|16;-9\|156;**-6\|413**;-3\|9;0\|140;**3\|365** |
| 12102 | 25\|26 | 25\|26 | NA | -9.59 | -9\|4;-6\|2;0\|6;3\|22;6\|135;**9\|351**;**12\|313**;15\|2 |
| 12113 | 18\|23 | 18\|23 | NA | -1.35 | -30\|2;-24\|2;-21\|3;-18\|20;-15\|149;**-12\|565**;-9\|8;-6\|10;-3\|36;0\|152;**3\|623**;6\|4 |
| 12338 | 21\|26 | 21\|26 | NA | -0.20 | -9\|6;-6\|36;**-3\|122**;6\|4;9\|54;**12\|115**;15\|2 |
| 12339 | 26\|29 | 26\|29 | NA | -1.11 | 3\|2;6\|9;9\|16;**12\|54**;15\|4;18\|16;**21\|39** |
| 12579 | 22\|23 | 22\|23 | 22 | -3.60 | -18\|2;-9\|4;-6\|12;-3\|62;**0\|228**;**3\|157**;6\|2 |
| 12789 | 23\|24 | 23\|24 | NA | -1.35 | -6\|2;-3\|6;0\|34;**3\|113**;**6\|114** |
| 14694 | 21\|22 | 21\|22 | NA | -9.00 | -21\|2;-15\|4;-9\|24;-6\|130;**-3\|474**;**0\|409**;3\|2 |
| 9071 | 19 | 19 | 19 | 0.00 | -15\|2;-12\|12;**-9\|84** |
| 9599 | 23 | 23 | NA | -1.19 | 0\|10;**3\|26** |
| 12186 | 25 | 25 | NA | -4.45 | 6\|9;**9\|14** |
| 5248 | 23 | 22\|23 | 23 | -75.52 | -18\|2;-15\|2;-12\|2;-9\|2;-6\|6;-3\|90;**0\|350**;**3\|1039**;6\|7;9\|2 |
| 6277 | 22 | 21\|22 | 22 | -108.16 | -21\|8;-9\|18;-6\|132;**-3\|502**;**0\|1510**;3\|4;15\|2 |
| 6723 | 26 | 25\|26 | 26 | -56.56 | -9\|6;-6\|2;-3\|4;0\|4;3\|6;6\|73;**9\|321**;**12\|889**;15\|8 |
| 6774 | 28 | 27\|28 | 28 | -57.55 | -3\|6;3\|2;6\|6;9\|26;12\|138;**15\|472**;**18\|1228**;21\|6 |
| 7033 | 24 | 23\|24 | 24 | -45.80 | -15\|4;-12\|4;-9\|4;-6\|13;-3\|18;0\|103;**3\|429**;**6\|1055**;9\|8 |
| 7064 | 21 | 20\|21 | 21 | -144.37 | -24\|6;-21\|2;-18\|4;-15\|2;-12\|6;-9\|132;**-6\|501**;**-3\|1722**;0\|16 |
| 7178 | 20 | 19\|20 | 20 | -95.43 | -24\|2;-18\|2;-15\|8;-12\|64;**-9\|249**;**-6\|956**;-3\|14 |
| 7314 | 26 | 25\|26 | 26 | -28.26 | -6\|2;0\|4;3\|14;6\|38;**9\|226**;**12\|541**;15\|4 |
| 7898 | 23 | 22\|23 | 23 | -76.20 | -18\|7;-12\|4;-9\|4;-6\|10;-3\|89;**0\|351**;**3\|1072**;6\|2;18\|1 |
| 9094 | 23 | 22\|23 | 23 | -64.19 | -18\|4;-15\|4;-12\|4;-9\|4;-6\|8;-3\|65;**0\|37**0;**3\|1023**;6\|2 |
| 9174 | 22 | 21\|22 | 22 | -169.31 | -21\|10;-18\|6;-12\|10;-9\|28;-6\|133;**-3\|665**;**0\|2092**;3\|16;9\|1 |
| 9196 | 25 | 24\|25 | 25 | -57.68 | -12\|6;-9\|2;-6\|2;-3\|8;0\|11;3\|89;**6\|369**;**9\|1005**;12\|8 |
| 9290 | 26 | 25\|26 | 26 | -64.71 | -9\|8;-6\|2;-3\|6;0\|6;3\|17;6\|89;**9\|503**;**12\|1205**;15\|8 |
| 9341 | 19 | 18\|19 | 19 | -39.69 | -30\|3;-18\|2;-15\|24;**-12\|116**;**-9\|422**;-6\|2;0\|2 |
| 9546 | 20 | 19\|20 | 20 | -91.84 | -27\|6;-15\|4;-12\|45;**-9\|298**;**-6\|979**;6\|2 |
| 9697 | 16 | 15\|16 | 16 | -184.74 | -36\|2;-33\|4;-30\|4;-27\|10;-24\|76;**-21\|375**;**-18\|1600**;-15\|6;0\|1 |
| 9728 | 21 | 20\|21 | 21 | -98.95 | -24\|2;-21\|2;-18\|4;-15\|6;-12\|11;-9\|95;**-6\|365**;**-3\|1241**;0\|10 |
| 9857 | 22 | 21\|22 | 22 | -73.46 | -15\|2;-12\|2;-9\|12;-6\|36;**-3\|211**;**0\|756** |
| 9913 | 19 | 18\|19 | 19 | -125.77 | -30\|5;-27\|2;-21\|2;-18\|8;-15\|94;**-12\|391**;**-9\|1423**;-6\|6;3\|2 |
| 10191 | 23 | 22\|23 | 23 | -57.46 | -18\|6;-9\|2;-6\|10;-3\|77;**0\|323**;**3\|902**;6\|12 |
| 10243 | 21 | 20\|21 | 21 | -126.99 | -24\|2;-15\|4;-12\|14;-9\|95;**-6\|462**;**-3\|1494**;0\|10;9\|2 |
| 10549 | 24 | 23\|24 | 24 | -51.34 | -15\|2;-3\|4;0\|32;**3\|154**;**6\|520** |
| 10771 | 21 | 20\|21 | 21 | -117.31 | -24\|4;-21\|2;-18\|4;-15\|8;-12\|16;-9\|74;**-6\|460**;**-3\|1463**;0\|10;12\|2 |
| 11186 | 25 | 24\|25 | 25 | -65.01 | -12\|4;0\|6;3\|68;**6\|226**;**9\|783**;12\|2 |
| 11311 | 23 | 22\|23 | 23 | -81.58 | -18\|10;-15\|4;-12\|2;-9\|8;-6\|10;-3\|99;**0\|462**;**3\|1315**;6\|14 |
| 11549 | 14 | 13\|14 | 14 | -inf | -36\|2;-30\|58;**-27\|497**;**-24\|2561**;-21\|2;-6\|1 |
| 11708 | 27 | 26\|27 | 27 | -44.57 | -6\|2;-3\|2;3\|4;6\|8;9\|94;**12\|363**;**15\|912**;18\|4 |
| 11745 | 13 | 12\|13 | 13 | -246.95 | -48\|2;-42\|2;-36\|10;-33\|42;**-30\|242**;**-27\|1499**;-24\|4 |
| 11806 | 23 | 22\|23 | 23 | -91.47 | -15\|4;-12\|2;-9\|5;-6\|24;-3\|106;**0\|520**;**3\|1433**;6\|2 |
| 11969 | 19 | 18\|19 | 19 | -108.43 | -30\|2;-27\|4;-24\|2;-18\|8;-15\|34;**-12\|254**;**-9\|995**;-6\|6;-3\|2;3\|2;9\|2 |
| 12009 | 24 | 23\|24 | 24 | -83.47 | -15\|8;-12\|4;-9\|4;-6\|1;-3\|14;0\|122;**3\|455**;**6\|1331**;9\|11 |
| 12025 | 25 | 24\|25 | 25 | -41.61 | -12\|2;-9\|4;-6\|4;-3\|7;0\|8;3\|74;**6\|315**;**9\|769**;12\|5 |
| 12141 | 22 | 21\|22 | 22 | -73.30 | -18\|2;-9\|4;-6\|74;**-3\|457**;**0\|1192**;3\|2;6\|2;18\|2 |
| 12253 | 21 | 20\|21 | 21 | -135.02 | -24\|5;-21\|2;-18\|2;-15\|4;-12\|18;-9\|86;**-6\|406**;**-3\|1470**;0\|2 |
| 12272 | 26 | 25\|26 | 26 | -50.66 | -9\|4;-3\|2;0\|2;3\|17;6\|108;**9\|398**;**12\|1051**;15\|6 |
| 12591 | 26 | 25\|26 | 26 | -92.50 | 0\|4;3\|12;6\|95;**9\|388**;**12\|1240**;15\|9 |
| 13186 | 24 | 23\|24 | 24 | -28.93 | -12\|2;-3\|4;0\|26;**3\|98**;**6\|326** |
| 14288 | 19 | 18\|19 | 19 | -39.32 | -21\|2;-18\|2;-15\|26;**-12\|111**;**-9\|423**;-6\|4;3\|2 |
| 14833 | 24 | 23\|24 | 24 | -7.11 | -3\|2;0\|6;**3\|16**;**6\|78** |

NA= Not Available. *= Samples with a heterozygous genotype identified by HipSTR and capillary electrophoresis and missed by Sanger. inf= mathematical infinity.

CAG repeats by capillary electrophoresis were computed considering that sequence reference, sizing 223 bp, corresponds with 22 CAG repeats.

MALLREADS indicates the base pairs differences from the reference allele and the number of reads reporting that differences, separated by the pipe character (|).
